# Biological and prognostic value of ETV5 in high-grade serous ovarian cancer

**DOI:** 10.1186/s13048-021-00899-6

**Published:** 2021-11-04

**Authors:** Lu Zhang, Ruiting Fu, Ping Liu, Lijun Wang, Weihua Liang, Hong Zou, Wei Jia, Lin Tao

**Affiliations:** 1 Department of Pathology, NHC Key Laboratory of Prevention and Treatment of Central Asia High Incidence Diseases/The First Affiliated Hospital, Shihezi University School of Medicine, Shihezi, 832003 China; 2 Department of Pathology, Shenzhen Traditional Chinese Medicine hospital, Shenzhen, 518033 China; 3 Department of Obstetrics and Gynecology, The First Affiliated Hospital School of Medicine, Shihezi University, Shihezi, 832003 China

**Keywords:** ETV5, High-grade serous ovarian cancer (HGSOC), Carcinogenesis, Biomarker, Prognosis

## Abstract

**Background:**

ETS transcription factors are known to act as either positive or negative regulators of the expression of genes involved in various biological processes. It was reported that ETS variant transcription factor 5 (ETV5), a key member of the ETS family, mainly plays a role as an potential oncogene in various malignant tumors. However, the role and mechanism of ETV5 in high-grade serous ovarian cancer (HGSOC) have not been elucidated.

**Methods:**

Quantitative real-time polymerase chain reaction (qRT-PCR) assay was used to detect ETV5 messenger ribonucleic acid (mRNA) expression in 87 HGSOC tissues and 35 normal fallopian tube tissues. Western blotting and qRT-PCR were used to detect the protein and mRNA expression of ETV5 in six ovarian cancer (OC) and human embryonic cell lines. Knockdown or overexpression of ETV5 in HGSOC cell lines, Cell Counting Kit-8, colony formation, and transwell assays were used to detect HGSOC cell proliferation, invasion, and migration capabilities. The chi-square test was used to analyze the clinicopathological characteristics of HGSOC patients. Survival analysis was performed using the Kaplan-Meier method, and the log-rank test was used to analyze the correlation between ETV5 expression and HGSOC patient prognosis. Univariate and multivariate analyses using the Cox regression model were conducted to determine the independent significance of relevant clinical covariates.

**Results:**

Bioinformatic analysis demonstrated that ETV5 expression was significantly upregulated in OC (*p* < 0.05). qRT-PCR showed that ETV5 was significantly overexpressed in HGSOC tissues than in fallopian tube tissues (*p* < 0.05). qRT-PCR and western blotting assays demonstrated that ETV5 was relatively highly expressed in OC cell lines. ETV5 overexpression was positively associated with poor survival in HGSOC patients, therefore making it a high-risk factor for HGSOC progression. Furthermore, ETV5 promoted the proliferation, migration, and invasion capabilities of HGSOC cells.

**Conclusion:**

ETV5 has a carcinogenic effect in HGSOC and can be used as a clinically effective biomarker to determine the prognosis of HGSOC patients.

**Supplementary Information:**

The online version contains supplementary material available at 10.1186/s13048-021-00899-6.

## Introduction

Ovarian cancer (OC) is one of the most lethal gynecological cancers [1]. In the United States, approximately 22,000 incident OC cases and 14,000 related deaths were projected in 2020 [2]. High-grade serous ovarian cancer (HGSOC) is the most common subtype of OC, accounting for 75% of all epithelial OCs [3]. Due to the asymptomatic nature of HGSOC and lack of effective biomarkers for early diagnosis, approximately 75% of patients are diagnosed at an advanced stage [4]. The 5-year relative survival rate of advanced-stage patients is only 29%, while that of early-stage patients is 92% [5]. Therefore, it is essential to explore new biomarkers for HGSOC carcinogenesis and prognosis, as well as alternative therapeutic targets.

ETS variant transcription factor 5 (ETV5), also known as ERM transcription factor, is a member of the ETS transcription factor family. The ETS family contains an evolutionarily conserved ETS domain that has 85 amino acids and binds to a DNA sequence with a 5′-GGA(A/T)-3′ core [6]; it plays an important role in various physiological processes, such as cell development, differentiation, proliferation, and apoptosis [7]. Transcriptome analysis demonstrated that ETV5 is widely expressed in multiple organs during embryonic development and adulthood [8]. During embryonic development, it is mainly involved in mouse embryonic mesokidney differentiation and the development of mammary duct branches [9]. Additionally, ETV5 is involved in the self-renewal of spermatogonial stem cells [10], and the maintenance of type II alveolar epithelial cells in the alveoli of adult mice [11].

Recent studies have found that ETV5 is overexpressed in several malignant tumors, such as OC, inducing transcription of multiple oncogenes involved in cell apoptosis resistance, angiogenesis, migration, invasion, and other malignant biological behaviors [12–14]. ETV5 is abnormally highly expressed in colorectal cancer (CRC) and promotes its angiogenesis [15]. In papillary thyroid carcinoma, ETV5 is upregulated and may be involved in its epithelial–mesenchymal transition (EMT) [16]. Similarly, ETV5 induces the epithelial–mesenchymal transformation of endometrial cancer through transcriptional activation of the nidogen-1 (NID1) and nuclear protein 1 (NUPR1) genes, participating in the process of endometrial cancer invasion [17]. In OC, ETV5 is highly expressed and promotes the apoptosis resistance of OC cells through transcriptional activation of the forkhead box protein M1 (FOXM1) gene [14]. However, the role of ETV5 in the development of HGSOC and the molecular mechanism underlying its involvement in carcinogenesis remains unclear.

Thus, this study aimed to investigate the influence of ETV5 on the prognosis and malignant biological behaviors of HGSOC. Towards this goal, ETV5 expression was detected in normal fallopian tubes, HGSOC tissues, and HGSOC cell lines.

## Materials and methods

### Study design and patients

This was a retrospective study of HGSOC patients who were treated at the First Affiliated Hospital of Shihezi University School of Medicine between 2010 and 2019. The clinicopathological data, including age, International Federation of Gynecology and Obstetrics (FIGO) stage, recurrence, chemotherapy response, ascites, disease-free survival, and overall survival, of these patients were collected from the on-paper medical records and electronic medical record system of the hospital. In total, 87 HGSOC tissues and 35 normal fallopian tube tissues were obtained. All tissues were fixed in 10% neutral formalin, processed, and embedded in paraffin. Diagnoses were confirmed through hematoxylin-eosin and immunohistochemical staining by two experienced pathologists, following the World Health Organization’s Pathology and Genetics Tumors of the Breast and Female Genital Organs (8th edition) guidelines.

This study was approved by the Ethics Committee of the First Affiliated Hospital of Shihezi University School of Medicine (Approval number: 2019–008-01) and was conducted according to the tenets of the Declaration of Helsinki. All patients provided written informed consent.

### Trial inclusion and exclusion criteria

#### The inclusion criteria

In the experimental group, HGSOC samples from patients who met the following conditions were included in the study:1. newly diagnosed HGSOC patients who had not undergone radiotherapy and chemotherapy, and 2. patients diagnosed with primary HGSOC. In the control group, Fallopian tube samples from patients who met the following conditions were included in the study:1. patients who had undergone hysterosalpingectomy due to benign disease, and 2. patients who had not undergone radiotherapy and chemotherapy before surgery.

#### The exclusion criteria

Patients were excluded from the study if they had undergone radiotherapy and chemotherapy before surgery, as well as cases where consent according to national regulations was not obtainable, when the investigator was unable to obtain the necessary consent from the patient before inclusion in the study.

### Bioinformatic analysis

Gene expression profile data (GSE18520, GSE36668, and GSE14407) were downloaded from the Gene Expression Omnibus (GEO) database (https://www.ncbi.nlm.nih.gov/geo/) (Affymetrix Human Genome U133 Plus 2.0 Array). GEO is a public functional genomics database that stores data on gene expression profile, methylation profile, chips, and microarrays. GSE18520 contains data from 53 OC and 10 normal ovarian surface epithelial samples. GSE36668 contains data on four serous OC and four normal ovarian epithelial samples. GSE14407 contains data on 12 OC and 12 normal ovarian surface epithelial samples. The GEO2R online tool was used to analyze the differentially expressed genes between OC tissues and normal tissues in the three datasets. A cutoff value of ∣log 2-fold change (FC)∣ > 2 (FC) and a *p*-value < 0.01 were set as the statistical significance threshold. Gene Expression Profiling Interactive Analysis (GEPIA), an online analysis tool based on data from The Cancer Genome Atlas (TCGA) database, was used to analyze ETV5 differential expression between OC and normal tissues (http://gepia.cancer-pku.cn/).

### Cell lines and antibodies

The human epithelial OC cell lines SKOV3, OV2008, A2780, HEYA8, OVCA433, and C13*, and the normal human ovarian epithelial cell line IOSE80 were cultivated in the Roswell Park Memorial Institute (RPMI-1640) medium. The human embryonic kidney cell line, 293 T, was cultivated in Dulbecco’s modified eagle medium (DMEM), supplemented with 10% fetal bovine serum (FBS) in a humidified tissue culture incubator containing 5% CO2 at 37 °C. SKOV3, HEYA8, and 293 T cells were purchased from the Chinese Academy of Sciences Type Culture Collection (Shanghai, China). OVCA433 and A2780 cells were a generous gift from Dr. Gang Cheng (Department of Gynecology and Obstetrics, Tongji Hospital of Huazhong University of Science and Technology). OV2008 and C13* cells were obtained from Prof. Benjamin K. Tsang (Ottawa Health Research Institute, Ottawa, Canada). The IOSE80 cell line was purchased from Shanghai Gaining Biotechnology Co., Ltd. (Shanghai, China).

### RNA extraction and quantitative real-time PCR

Total RNA was extracted from OC cell lines using the miRNeasy Mini Kit (Qiagen, Valencia, CA, USA) and from HGSOC and fallopian tube tissues using the miRNeasy FFPE Kit (Qiagen, Valencia, CA) in accordance with the manufacturer’s instructions. Complementary deoxyribonucleic acid (cDNA) was synthesized using the QuantiTect Reverse Transcription Kit (Qiagen). Quantitative real-time polymerase chain reaction (qRT-PCR) was performed on a 7500 Fast Real-Time PCR System (Life Technologies, Shanghai, China) using a QuantiFast SYBR Green PCR kit (Qiagen) in accordance with the manufacturer’s instructions. The primers used in this study were prepared by the Shanghai Sangon Biotechnology Company. The primer sequences are listed in Table 1.

**Table 1:** qRT-PCR relatively primer sequences

Genes	forward primer(5′-3′)	reverse primer(5′-3′)
ETV5	CAGCACACGGGTTCCAGTCAC	TGGCAGTTAGGCACTTCTGAATCG
GAPDH	GAGTCAACGGATTTGGTCGT	TTGATTTTGGAGGGATCTCG

### Western blotting assay

Western blotting was performed according to standard protocols. The primary antibodies used in this study included mouse anti-human monoclonal ETV5 antibody (1:1000 dilution; Biorbyt, UK) and mouse anti-human monoclonal beta (β)-actin antibody (1:1000 dilution; Zhongshan Biotechnology, Beijing, China). Goat anti-rabbit/mouse immunoglobulin G conjugated to horseradish peroxidase (IgG-HRP), used as the secondary antibody, was purchased from Zhongshan Biotechnology (Beijing, China). Protein signals were detected using an enhanced chemiluminescence kit (Thermo, Waltham, MA, USA). β-actin was used as an internal control.

### Proliferation assay

SKOV3, A2780, and OV2008 cells were incubated and transfected with the different treatments for 48 h, and the Cell Counting Kit-8 (CCK-8) assay was performed to construct a cell growth curve. The optical density (OD) value was determined by measuring the absorbance at 450 nm with a microplate reader at the same time every day until the seventh day, from the beginning of cell attachment. The daily OD values of each group of cells were used to generate a cell growth curve.

### Colony formation assay

The effect of ETV5 silencing on the proliferation of A2780 and SKOV3 cells and of ETV5 overexpression on the proliferation of OV2008 cells was analyzed using clone formation assays. Approximately 800 A2780, SKOV3, and OV2008 cells were cultured in a 6-well plate containing 10% FBS and in the RPMI-1640 medium for 10 days at 37 °C with 5% CO2. The colonies were washed twice with 1× phosphate-buffered saline (PBS), fixed for 5 min with 4% paraformaldehyde, and stained for 20 min (0.1% crystal violet staining solution, Beyotime, China). The number of colonies with more than 50 cells in a low-magnification visual field was counted, and the colony formation rate was calculated using the following formula: clone formation rate = (number of clones/seeded cells) × 100%.

### Migration and invasion assays

The cell migration and invasion assays were performed using a 24-well Transwell chamber (Corning Incorporation, Corning, NY, USA). Cells were seeded at a density of 4 × 104 cells into the upper chamber (pore size, 8 μm), the lower chamber was filled with 600 μl RPMI-1640 medium containing 10% FBS. For invasion assays, 1 × 105 cells in serum-free medium were placed into the upper chamber of an insert coated with Matrigel. Medium containing 10% FBS was added to the lower chamber. Following a 24 h incubation period at 37 °C (invasion assay incubating for 48 h), the cells remaining on the upper membrane were removed with cotton wool. Cells that had migrated or invaded through the membrane were fixed with methanol, stained with 0.1% crystal violet, imaged, and counted using an inverted microscope (Olympus, Tokyo, Japan).

### Statistical analysis

Between-group differences were assessed using a paired two-tailed Student’s t-test. The chi-square test was used to analyze the clinicopathological features associated with ETV5 expression in HGSOC. Survival curves were generated using the Kaplan–Meier survival plots and were compared using log-rank tests. Univariate and multivariate Cox regression models were used to determine the effects of variables on survival. All statistical analyses were performed using SPSS software (version 22.0; IBM Corp., Armonk, NY, USA). A *p*-value < 0.05 was considered statistically significant.

## Results

### ETV5 expression in HGSOC cells and tissues

**Fig. 1:**
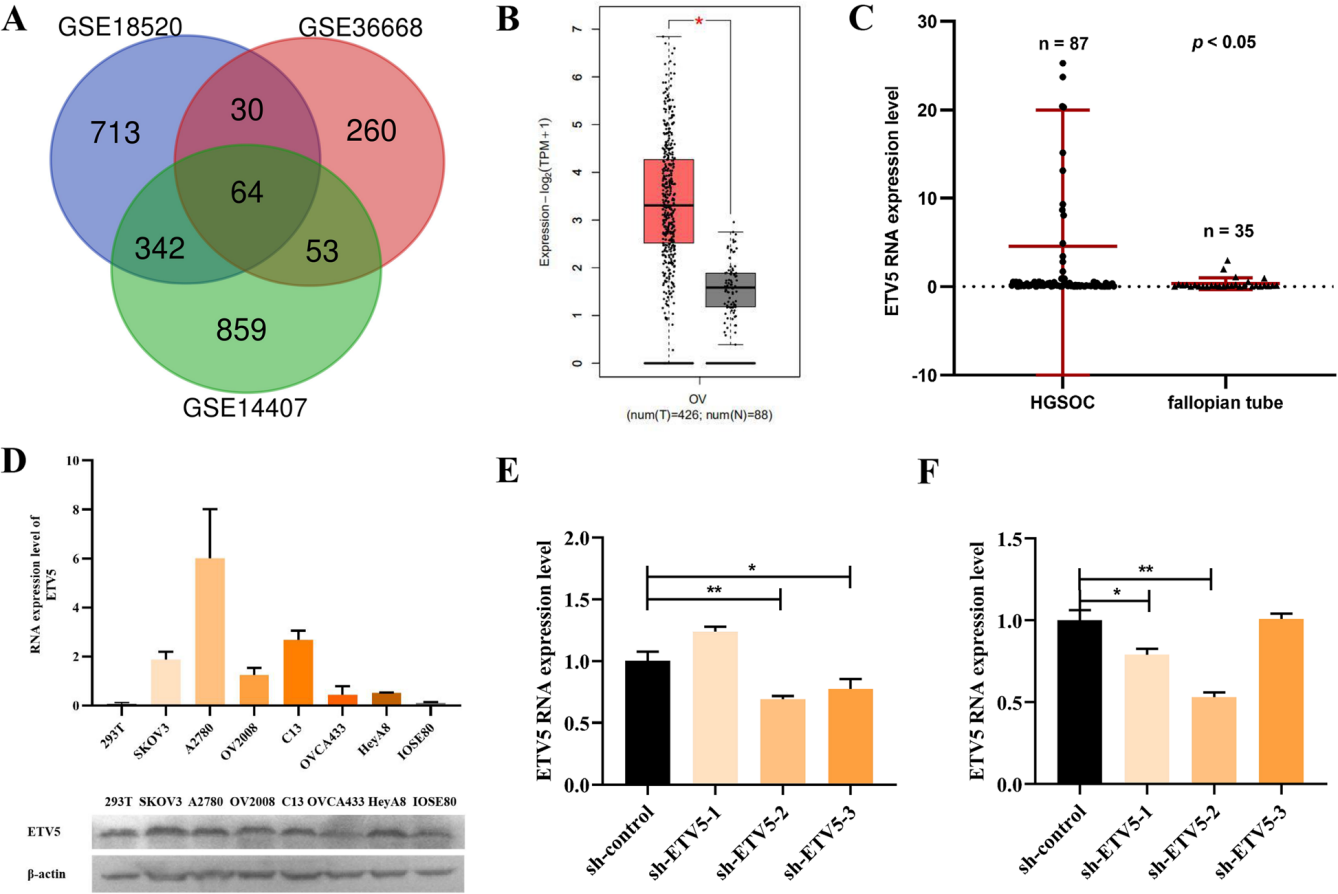
ETV5 was highly expressed in HGSOC tissues and cell lines. (**A**) Overall, 64 differentially expressed genes between ovarian carcinoma and normal tissues were screened from 3 datasets. (**B)** In GEPIA, ETV5 was highly expressed in ovarian carcinoma compared to that in normal tissues (*p* < 0.05). (**C**) ETV5 was significantly overexpressed in HGSOC tissues compared to that in normal fallopian tube tissues (*p* < 0.05). (**D**) ETV5 was relatively high expressed in HGSOC cell lines. Three ETV5-knockdown plasmids were transfected in SKOV3 (**E**) and A2780 (**F**) cell lines to screen out the plasmid with the best knockdown efficiency-sh-ETV5–2. *p < 0.05, **p < 0.01, ***p < 0.001

The differentially expressed genes between OC and normal tissues were analyzed separately in three datasets from the GEO database. Genes with a > 2-fold change and p-value < 0.01 were considered discriminatively expressed. The results demonstrated that 64 genes were upregulated in OC tissues, and ETV5 was identified as a candidate (see Fig. 1A). Moreover, differential expression analysis from TCGA data revealed that ETV5 was overexpressed in OC tissues than in normal tissues (*p* < 0.05, see Fig. 1B). qRT-PCR was performed to detect ETV5 expression between HGSOC tissues and normal fallopian tube tissues, and qRT-PCR and western blotting were performed to detect the expression of six HGSOC cell lines and human embryonic kidney cell line, 293 T. The results revealed that ETV5 was upregulated in HGSOC tissues than in normal fallopian tube tissues (*p* < 0.05, see Fig. 1C). Compared to the 293 T cell line, ETV5 was relatively highly expressed in six HGSOC cell lines. Specifically, ETV5 showed high endogenous expression in SKOV3 and A2780 cell lines and low endogenous expression in OV2008 cell lines. SKOV3 and A2780 cell lines with high endogenous expression of ETV5 were transfected with ETV5-knockdown plasmid (sh-ETV5–2), whereas OV2008 cell lines with low endogenous ETV5 expression were transfected with ETV5-overexpressed plasmid (ETV5) (see Fig. 1D). Moreover, qRT-PCR was used to verify the knockdown effect of three ETV5-knockdown plasmids and showed that sh-ETV5–2 exhibited the most significant knockdown effect (*p* < 0.01, see Fig. 1E, F).

### Correlation of ETV5 expression with clinicopathological parameters and survival

To explore the clinical value of ETV5 in HGSOC patients, we analyzed the association between ETV5 expression and several clinicopathological parameters. ETV5 overexpression was associated with age (*p* < 0.05), FIGO stage (*p* < 0.05), and chemotherapy response (*p* < 0.05). No significant correlations were observed between ETV5 expression and other clinicopathological parameters, including recurrence and ascites (see Table 2).

**Table 2:** ETV5 expression and clinicopathological parameters

Characteristies	n	ETV5 expression		χ2	*P*
	87	Over expression (%)	Low expression (%)		
Age					
< 55	44	20(45.5)	24(54.5)	4.587	0.032**
≥55	43	32(74.4)	11(25.6)		
FIGO stage					
I-II	21	12(57.1)	9(42.9)	4.212	0.040*
III-IV	66	40(60.6)	26(39.4)		
Chemotherapy response					
sensitive	26	10(38.5)	16(61.5)	6.502	0.011*
partial	24	16(66.7)	8(33.3)		
unknown	37				
Recurrence					
No	27	14(51.9)	13(48.1)	0.979	0.322
Yes	10	7(70.0)	3(30.0)		
unknown	50				
Ascites					
No	26	15(57.7)	11(42.3)	0.201	0.654
Yes	61	32(52.5)	29(47.5)		

**Fig. 2:**
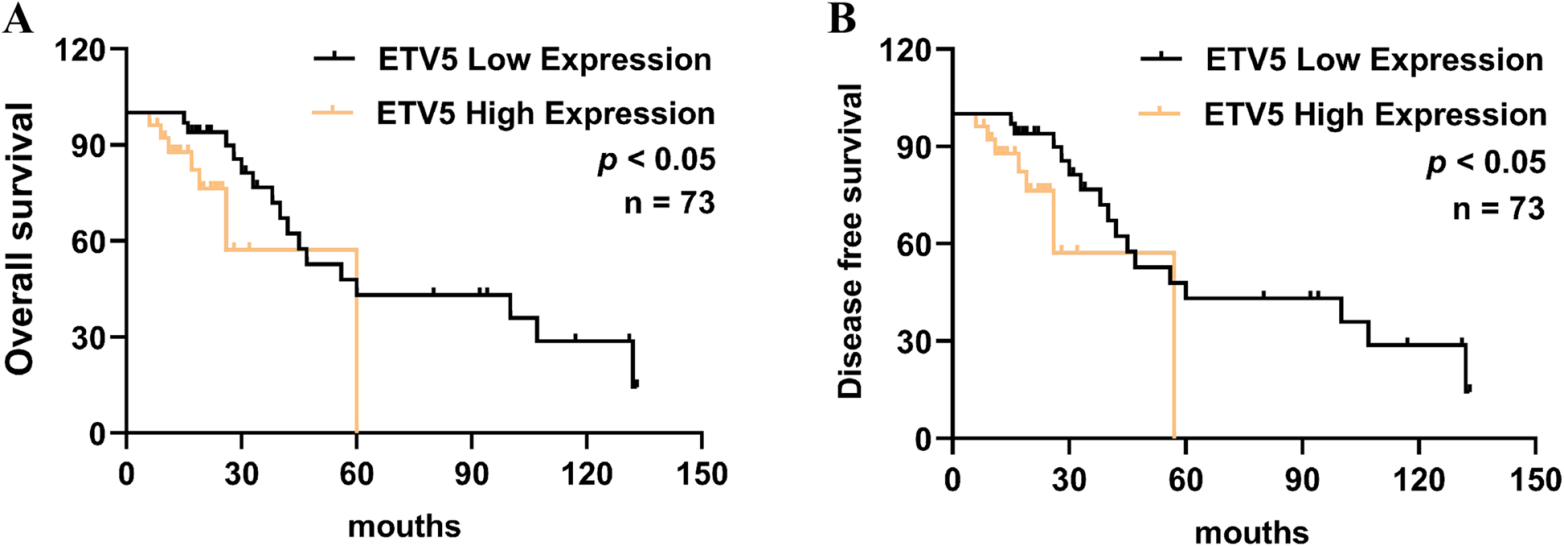
ETV5 is associated with a poor prognosis of HGSOC patients. In comparison to patients with low ETV5 expression, those with high expression has significantly lower (**A**) overall survival (OS) (*p* < 0.05) and (**B**) disease-free survival (DFS) (*p* < 0.05)

To investigate the prognostic significance of ETV5 overexpression, the Kaplan-Meier survival analysis was performed, and the results demonstrated that ETV5 overexpression was associated with poor overall survival (OS) (*p* < 0.05, see Fig. 2A) and disease-free survival (DFS) (*p* < 0.05, see Fig. 2B) in HGSOC patients. Furthermore, univariate Cox regression analysis revealed that ETV5 overexpression (*p* < 0.05), age (*p* < 0.05), recurrence (*p* < 0.05), and FIGO stage (*p* < 0.001) were associated with a shorter OS. Multivariate analysis identified recurrence (*p* < 0.01) and FIGO stage (*p* < 0.05) as independent prognostic factors in HGSOC patients (see Table 3).

**Table 3:** COX regression analysis of risk factors in patients with high-grade serous ovarian carcinoma

	Univariate analysis		Multivariate analysis	
	HR (95%CI)	*P* value	HR (95%CI)	*P* value
ETV5 expression (low- vs over-expression)	2.108(1.190–3.736)	0.011*	1.427(0.485–4.192)	0.518
Age (<55 vs ≥ 55)	0.534(0.322–0.885)	0.015*	0.713(0.379–1.340)	0.293
FIGO stage (I-IIvsIII-IV)	9.626(3.722–24.899)	≤0.001***	5.301(1.564–1.973)	0.007**
Recurrence (no vs yes)	2.542(1.178–5.482)	0.017*	2.670(1.140–6.253)	0.024*
Chemotherapy response (sensitive vs partial)	0.967(0.520–1.798)	0.915		
Ascites (no vs yes)	1.192(0.562–2.527)	0.647		

### Influence of ETV5 on HGSOC cell proliferation, migration, and invasion

**Fig. 3:**
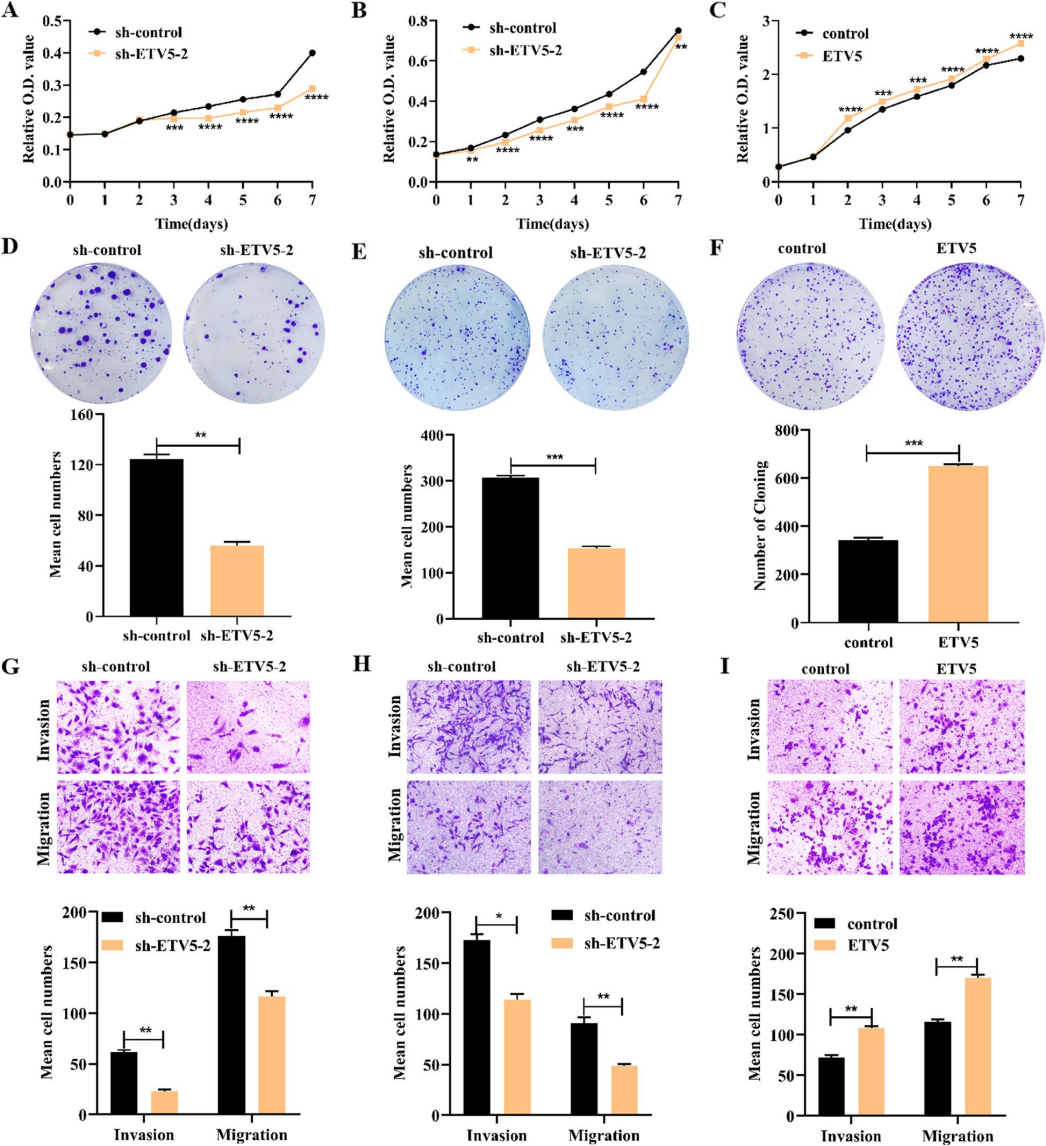
ETV5 promotes HGSOC cell proliferation, invasion, and migration in vitro. Cell proliferation is determined using the CCK8 assay after SKOV3 (**A**) and A2780 (**B**) cells are transfected with sh-ETV5–2 plasmid and OV2008 (**C**) cells are transfected with ETV5 plasmid. Clonality is detected using the colony formation assay after SKOV3 (**D**) and A2780 (**E**) cells are transfected with sh-ETV5–2 plasmid and OV2008 (**I**) cells. * p < 0.05, ** p < 0.01, ***p < 0.001, ****p < 0.0001

To investigate the function of ETV5 regarding HGSOC biological behaviors, we performed cell proliferation, invasion, and migration assays. SKOV3 and A2780 cell lines with high endogenous expression of ETV5 were transfected with sh-ETV5–2, whereas OV2008 cell lines with low endogenous ETV5 expression were transfected with ETV5-overexpressed plasmid (ETV5). The CCK-8 assay showed that, compared to negative controls, ETV5 markedly increased cell proliferation capability from 1 to 7 days (see Fig. 3A, B). In contrast, the proliferation of cells transfected with sh-ETV5–2 was significantly reduced (see Fig. 3C). The colony formation assay demonstrated that clone formation in SKOV3 and A2780 cell lines was diminished upon inhibition of ETV5 (p  < 0.01 and p  < 0.001, respectively, see Fig. 3D, E), while ETV5 promoted the formation of OV2008 cell clones (p < 0.001, see Fig. 3F). Furthermore, we examined the effect of ETV5 on cell invasion and migration by performing transwell assays. With this treatment, the invasive and migratory activities of SKOV3 (p < 0.01 and p < 0.01, respectively, see Fig. 3G) and A2780 (p < 0.05 and p < 0.01, respectively, see Fig. 3H) cell lines were suppressed compared to the negative control cell lines. Meanwhile, these phenotypes were enhanced in OV2008 cell lines (p  < 0.01 and p < 0.01, respectively, see Fig. 3I). These results indicate that ETV5 is involved in the regulation of cell proliferation, invasion, and migration.

## Discussion

Despite recent advances in treatment, including the introduction of targeted therapy and immunotherapy, HGSOC patients continue to have a poor clinical treatment benefit and prognosis. This is mainly owing to the unclear pathogenesis, high heterogeneity, low early diagnosis rate, and poor treatment effect for drug-resistant relapses in HGSOC. Therefore, it is crucial to explore the mechanism of HGSOC invasion and metastasis and identify the key molecules involved in its malignant transformation to establish potential therapeutic targets. The results of this study showed that ETV5 expression was significantly higher in OC tissues than in normal tissues.

Transcription factors are a set of sequence-specific DNA-binding proteins that regulate gene transcription by binding to specific double-stranded DNA sequences [18]. They can be combined with the promoter regions of target genes and recruit transcriptional regulatory proteins, such as ribonucleic acid (RNA) polymerase II, to form transcription initiation complexes, thereby initiating the transcription of target genes. In this study, ETV5, which is abnormally highly expressed in HGSOC, was screened using bioinformatics analysis. ETV5 is located in the q27-q29 region of chromosome 3. It is a member of the polyomavirus enhancer activator 3 (PEA3) subgroup of the ETS transcription factor family, which is characterized by being evolutionarily conserved and capable of binding to DNA [6]. The interference of ETS transcription factor activity can lead to tumor occurrence, progression, and metastasis. The PEA3 subgroup contains three transcription factors: ETV1 (ER81), ETV4 (PEA3/E1AF), and ETV5 (ERM) [7]. Studies have shown that ETV1, ETV4, and ETV5 are overexpressed in a variety of tumors [19–21]. The high level of expression of these transcription factors usually leads to more aggressive and drug-resistant tumors.

Since the late 1990s, the fallopian tube has been recognized as the main site of origin for HGSOC [22]. In this study, ETV5 was abnormally upregulated in HGSOC tissues than in fallopian tube tissues. Consistent with our research results, ETV5 is overexpressed in various malignant tumors, including OC, and involved in tumor cell angiogenesis [15], migration [16], invasion [17], and other malignant biological behaviors, suggesting that ETV5 may serve as a potential molecular therapeutic target for HGSOC. Presently, ETV5 and its subfamily members are being used as molecular targets for clinical treatment studies. In breast cancer, the selective estrogen receptor modifier, tamoxifen, inhibits the expression of ETV4 and ETV5 messenger ribonucleic acid (mRNA), which could reduce the risk of breast cancer [23]. Additionally, in prostate cancer, BRD32048 is a new drug used for the treatment of ETV1-positive tumors by directly binding to ETV1 to inhibit its transcriptional activity [24]. Therefore, through in-depth research, ETV5 is also likely to become a molecular target for targeted therapy of HGSOC in the future.

We further analyzed the correlation between ETV5 mRNA expression and prognosis as well as the clinicopathological factors in HGSOC patients. The OS and DFS of patients with ETV5 overexpression were significantly lower than in those with low expression. Regarding the clinical significance of ETV5 expression in HGSOC, we found that ETV5 overexpression was correlated with a high FIGO stage and chemotherapy resistance, indicating that it may be related to HGSOC progression. Univariate Cox regression analysis showed that high ETV5 expression, age, recurrence, and high FIGO stage were risk factors for HGSOC progression. As a member of the PEA3 subfamily of the ETS family, ETV5 and other PEA3 subfamily members play a carcinogenic role in tumors and are independent poor prognostic factors. Segalés L et al. reported that ETV1 transcription factor is highly expressed in prostate cancer, and that high mRNA level of ETV1 is associated with the short recurrence time of prostate-specific antigen [19]. In CRC, the expression of ETV4 mRNA is significantly high, and it is significantly positively correlated with the depth of invasion, lymph node metastasis, and tumor progression, suggesting that ETV4 can be used as a prognostic marker for CRC progression and metastasis [20, 25]. As an oncogenic transcription factor, ETV5 has been reported to be an independent predictor of the prognosis of patients with liver cancer [26]. Additionally, in triple-negative breast cancer [27, 28], gastric cancer [29], lung cancer [30], oral squamous cell carcinoma [31], and glioblastoma [32], the mRNA of ETV5 and its subfamily members were found to be highly expressed, and this was positively correlated with a high FIGO stage, depth of invasion, lymph node metastasis, and recurrence, leading to shorter survival time. The abovementioned studies suggest that ETV5 is related to the poor prognosis of a variety of solid tumors, and it may become a molecular marker for predicting the prognosis of patients with HGSOC.

Interestingly, immunohistochemistry, used to detect the protein expression of ETV5, showed no significant difference in the positive rate of ETV5 between HGSOC tissues (66.7%) and fallopian tube tissues (61.5%) (Additional file 1). Similar results were obtained with staining intensity. ETV5 expression in HGSOC tissues differs at the protein and mRNA levels. Qi et al. reported that ETV5 contains post-translational phosphorylation and ubiquitination sites [33]. Thus, we speculate that ETV5 is regulated by post-transcriptional or post-translational modifications, such as ubiquitination modification, resulting in inconsistent protein and mRNA expression in HGSOC. This needs to be explored in future studies.

Finally, we explored the effect of ETV5 on the malignant biological behavior of HGSOC. The results showed that ETV5 can promote the proliferation, migration, and invasion of HGSOC cells and that it has potential cancer-promoting effects. ETV5, consistent with other ETS family members, is an oncogenic transcription factor, involved in regulating multiple processes in tumor progression and metastasis, such as EMT, invasion, migration, cell cycle, and apoptosis, and chemotherapy resistance. In papillary thyroid carcinoma, ETV5 is highly expressed and can promote the expression of the twist-related protein 1 (TWIST1) gene, which has been shown to induce the EMT process [16]. In endometrial cancer, ETV5 could promote the invasion potential of tumor cells by up-regulating the expression of the zinc finger E-box binding homeobox 1 (ZEB1) gene and down-regulating the expression of epithelial (E)-cadherin [13]. In breast cancer, ETV4 down-regulates the expression of the cyclin D2 gene [34] and up-regulates the expression of the cyclin D3 gene [35] to promote cell proliferation. The overexpression of ETV1 in gastrointestinal stromal tumors can increase the expression of anti-apoptotic proteins, Bcl-2, and decrease the expression of pro-apoptotic proteins, Bax, thereby inhibiting tumor cell apoptosis [36]. In summary, ETV5 and its ETS family members can directly participate in the occurrence and development of tumors, or participate in tumor progression and metastasis by regulating the expression of key molecules involved in the process of tumor malignant transformation. However, further investigations in vitro and in vivo should be conducted to explore the downstream gene of ETV5 and further molecular mechanisms in carcinogenesis of HGSOC.

## Conclusions

In summary, our study provides new insights into the carcinogenic mechanism of HGSOC and identifies a candidate molecular marker for HGSOC prognosis and targeted therapy.

## Supplementary Information

**Additional file 1.** ETV5 protein expression of HGSOC and fallopian tube tissues.

## Data Availability

The datasets used and/or analyzed during the current study are available from the corresponding author upon reasonable request.

## Abbreviations

ETV5: ETS variant transcription factor 5
HGSOC: High-grade serous ovarian cancer
qRT-PCR: quantitative real-time polymerase chain reaction
OC: ovarian carcinoma
FIGO: International Federation of Gynaecology and Obstetrics
GEO: Gene Expression Omnibus
GEPIA: Gene Expression Profiling Interactive Analysis
TCGA: The Cancer Genome Atlas
