# Supplementary material for: Biological and prognostic value of ETV5 in high-grade serous ovarian cancer
Source: J Ovarian Res. 2021 Nov 4;14:149. doi: 10.1186/s13048-021-00899-6 (PMC8570011; doi:10.1186/s13048-021-00899-6)
Supplement: Supplementary file 1 — Additional file 1. ETV5 protein expression of HGSOC and fallopian tube tissues. [file 13048_2021_899_MOESM1_ESM.pdf]

ETV5 protein expression of HGSOC and fallopian tube tissues

HGSOC tissue

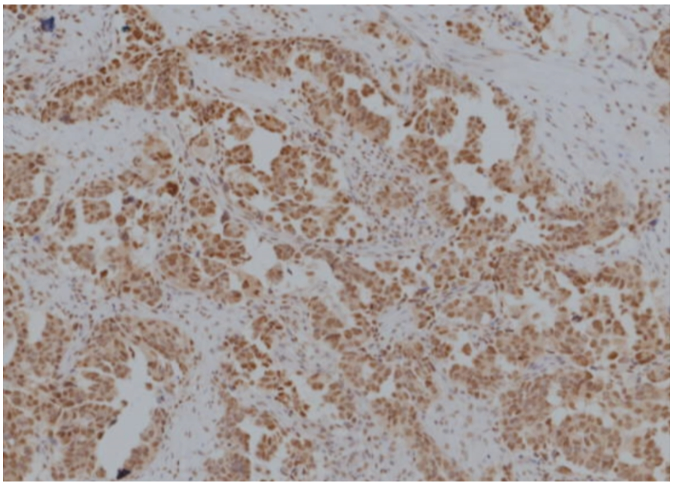

40×

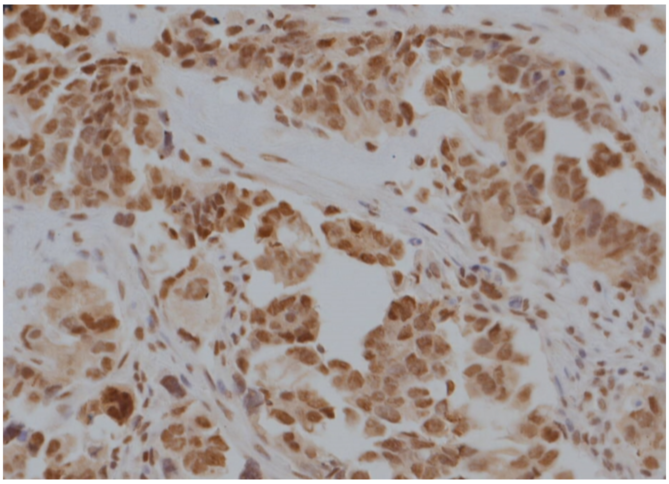

100×

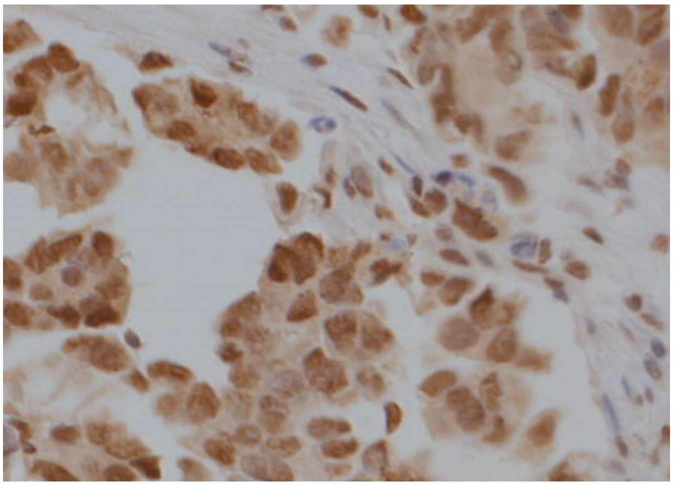

200×

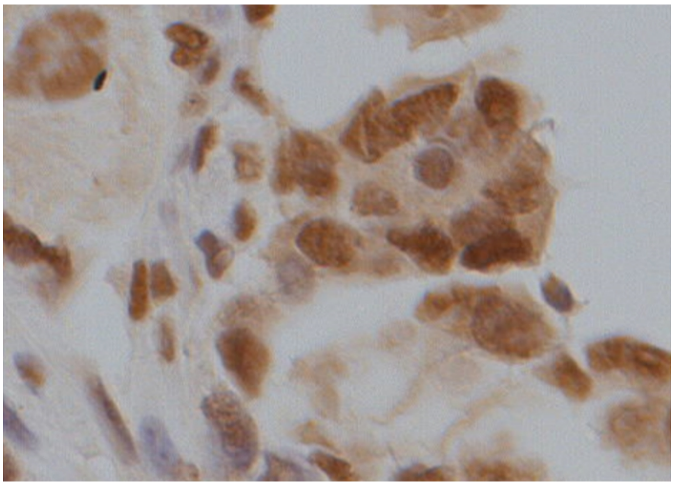

400×

Fallopian tube tissue

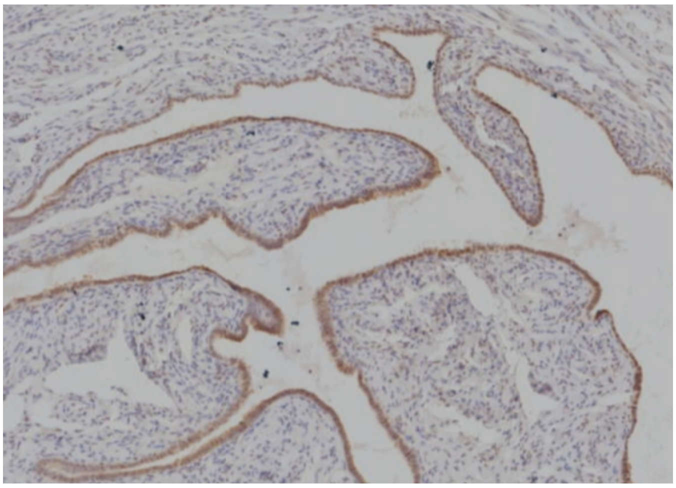

40×

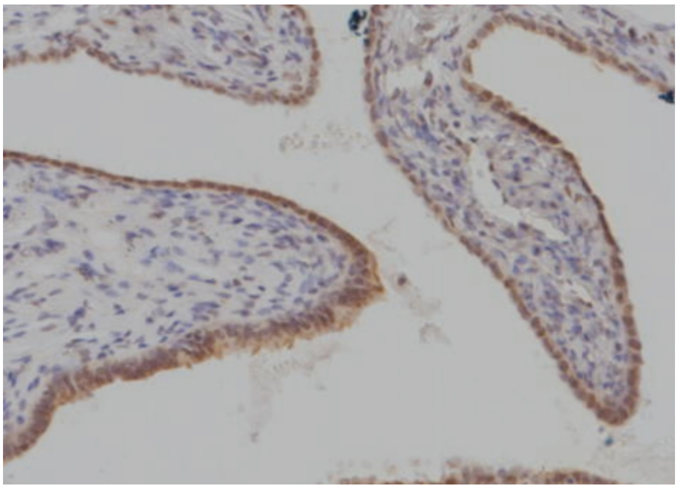

100×

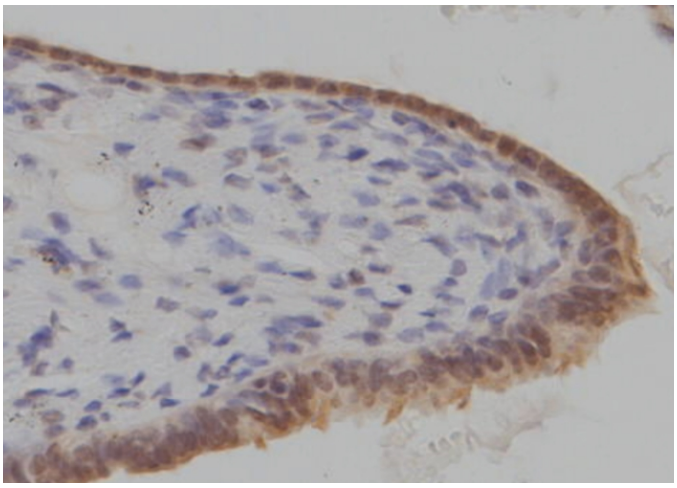

200×

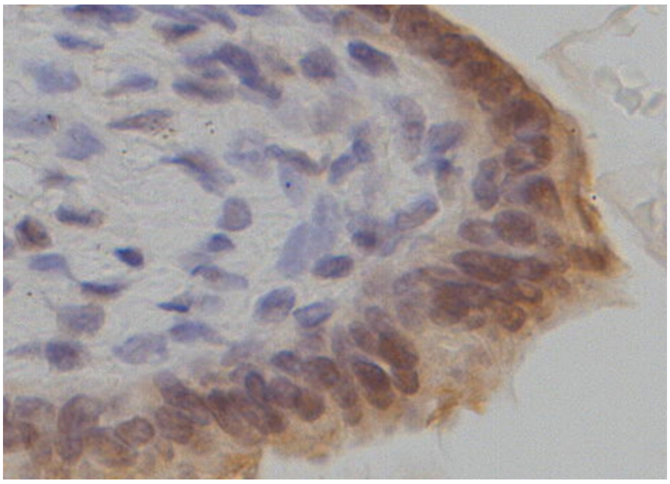

400×
